# Analysis of factors influencing the assessment of sexual self-defense capacity in individuals with mental disorders

**DOI:** 10.3389/fpsyt.2025.1714391

**Published:** 2025-12-03

**Authors:** Zhong Zuo, Qiang Wang, Jie Xu, Li Yuan, Zitong Shen, Xuedong Wang, Yunge Zhang, Jie Xu, Guangjian Li, Xianhu Yao, Yajun Xu

**Affiliations:** 1 Department of Psychology, Wannan Medical College, Wuhu, China; 2 School of Law, Nanjing University, Nanjing, China; 3 Forensic Psychiatric Examination Department, Forensic Appraisal Center of Beijing Borui Testing Co., Ltd., Beijing, China; 4 Guangyuan Jingwei Institute of Forensic Medical Appraisal, Guangyuan Mental Health Center, Guangyuan, China; 5 Suzhou Second People’s Hospital, Suzhou, China; 6 Anqing Sixth People’s Hospital, Anqing, China; 7 Psychology Department, Changzhou De’an Hospital, Changzhou, China; 8 Ma’anshan Fourth People’s Hospital, Ma’anshan, China

**Keywords:** forensic psychiatry, sexual self-defense capacity, mental disorders, intellectual level, activities of daily living, individuals with mental disorders

## Abstract

**Background:**

China’s Sexual Self-Defense Capacity (SSDC) assessment evaluates mentally disordered females’ ability to protect sexual rights, critically impacting rape adjudication. Despite 3-5× higher sexual assault risk in this population, key SSDC impairment predictors—Intellectual Level (FSIQ/VIQ/PIQ) and Activities of Daily Living (ADL)—face standardization challenges due to regional variations in assessment tools/thresholds.

**Methods:**

Retrospective analysis of 398 forensic SSDC cases (2020-2024) from five Chinese institutions, following national guidelines. Univariate, correlation, regression, ROC, and combined modeling analyses identified predictors.

**Results:**

(1) ADL score exerted the strongest effect on CSSAS, followed by FSIQ. (2) Critical thresholds established: Retained SSDC (CSSAS score≥21): ADL score ≤ 22.5 or FSIQ≥66.5; Diminished SSDC score(7≤CSSAS<21): 22.5<ADL score ≤ 31.5 or 53.5≤FSIQ<66.5; Absent SSDC (CSSAS score<7): ADL score>31.5 or FSIQ<53.5(3) Combined model (ADL score+FSIQ) achieved higher AUC than single factors.

**Conclusion:**

(1) Both ADL and FSIQ are significant factors influencing SSDC. (2) ADL demonstrates superior predictive efficacy compared to FSIQ. (3) The combined predictive model (ADL + FSIQ) outperforms the use of FSIQ alone. (4) The predictive efficacy of the combined ADL-FSIQ model failed to exceed that of the ADL-only model. (5) The comprehensive use of ADL and FSIQ assessments can effectively improve the accuracy of SSDC evaluation in forensic psychiatric expertise, facilitating rape identification and sentencing. It also helps law enforcement preliminarily judge the SSDC of involved individuals, guide targeted criminal investigations, and initiate forensic procedures promptly.

## 1 Introduction

“Sexual Self-Defense Capacity(SSDC)”:An individual’s ability to recognize, resist, and seek help in sexual assault situations, influenced by cognitive, psychological, and social factors (1). While the assessment of SSDC may include both male and female subjects in some jurisdictions, in China, it is exclusively applied to female examinees. Conceptually, SSDC refers to a female examinee’s capacity to comprehend and uphold her right to the inviolability of her sexual rights (2). This concept is intrinsically linked to the establishment of rape in sexual assault cases and serves as a crucial factor for criminal investigation and sentencing considerations in rape proceedings (3). On one hand, the assessment of SSDC provides a scientific basis for determining the victim’s capacity for sexual consent and evaluating the reliability of her testimony, thereby assisting investigative authorities in accurately determining the facts and nature of the case. On the other hand, it furnishes the judiciary with an objective standard for considering the victim’s particular vulnerability during sentencing, ensuring that punishment corresponds to culpability (4).

Both the World Health Organization (WHO) and the United Nations Convention on the Rights of Persons with Disabilities emphasize the protection of sexual autonomy for persons with mental disorders (5). In the United States, there is no unified standard for determining SSDC across states. Generally, a medical evaluation of the individual’s mental state is required, followed by a judicial determination based on the case specifics regarding their capacity to comprehend the nature and consequences of the sexual act and uphold their rights. This process also considers evidence such as the individual’s words and actions during the incident and testimony from expert witnesses (6). The US approach to SSDC assessment increasingly reflects a “victim-centered approach”, with the recent focus shifting from “whether resistance occurred” to “whether genuine consent existed,” signifying enhanced protection for sexual autonomy (7). Germany’s revised Section 177 of the Criminal Code (Strafgesetzbuch) in 2016 (8) introduced the “no-means-no” model, replacing the previous requirement of “force” as a necessary element for rape and other sexual offenses. It also added provisions criminalizing sexual harassment and offenses committed by groups.

In China, SSDC assessment is conducted according to the Guidelines for Assessment of SSDC in the Mentally Disordered (SF/T 0071-2020). These guidelines categorize SSDC into three levels: “Absent”, “Diminished”, and “Retained”. The assessment criteria comprise both medical and jurisprudential components. The medical criterion requires a formal diagnosis of a mental disorder in the examinee. The jurisprudential criterion evaluates whether, and to what extent, the mental disorder impairs the examinee’s capacity to comprehend and uphold her right to the inviolability of her sexual rights (2). The former constitutes the foundation for SSDC assessment, while the latter provides the guiding principle. As noted, SSDC assessment practices in China and internationally share a common focus on protecting the sexual autonomy of persons with mental disorders. However, differences exist in assessment procedures due to variations in cultural backgrounds and legal systems (9, 10).

Recent research indicates that individuals with severe mental disorders face a 2.5 times higher risk of criminal victimization compared to the general population (11). Their risk of experiencing violent crime is over four times higher, while the risk for specific crimes such as sexual assault and robbery ranges from 6 to 23 times higher (12). An epidemiological survey by Khalifeh et al. (2015) found that the prevalence of sexual assault among individuals with mental disorders is 3 to 5 times higher than in the general population (13). Furthermore, research indicates that women with disabilities are approximately twice as likely to experience sexual violence in their lifetime compared to women without disabilities (14). Individuals with mental disorders face heightened vulnerability due to the influence of psychotic symptoms (15). When sexually assaulted, the perpetrator is often an acquaintance. Additionally, the reporting rate among victims with mental disorders is lower than among the general population, a phenomenon strongly linked to cognitive impairments and inadequate social support (4). Consequently, Activities of Daily Living (16, 17) and Intellectual Level (18) are key predictive factors for SSDC. Intellectual disability and impaired ADL significantly diminish an individual’s cognitive understanding of sexual assault (7) and their capacity for resistance (19). In Chinese SSDC assessment practice, the ADL scale and the Wechsler Adult Intelligence Scale (WAIS) are commonly used instruments for evaluating examinees’ Intellectual Level and daily living functioning. However, variations in critical values for SSDC determination persist across different regions (20).

In summary, this study focuses on examinees involved in SSDC forensic assessments as its research subjects. It aims to investigate the factors influencing SSDC and attempts to predict the corresponding Intellectual Level and ADL profiles associated with different levels of SSDC. The findings are intended to provide a theoretical and practical foundation for the standardization of SSDC assessment methodologies, thereby promoting the development of scientifically rigorous and standardized forensic practices in sexual assault cases.

## 2 Methods

### 2.1 Study subjects

This study retrospectively analyzed 398 cases involving SSDC assessments conducted between 2020 and 2024 across five judicial appraisal institutions in China: Beijing Borui Testing Co., Ltd. Judicial Appraisal Center, Sichuan Guangyuan Jingwei Forensic Appraisal Institute, Anhui Lüyuan Judicial Appraisal Institute, Anhui Huaihai Judicial Appraisal Institute, and Anhui Huatai Judicial Appraisal Institute. From an initial pool of 417 legally appraised cases, 19 were excluded for failing to meet study criteria. Inclusion required: (1) involvement in alleged rape incidents; (2) a formal diagnosis of mental disorder meeting both ICD-10 (21) and CCMD-3 (22) criteria; and (3) unanimous appraisal conclusions regarding SSDC. Exclusion criteria encompassed: (1) non-cooperation from the examinee; (2) insufficient, unreliable, or incomplete appraisal materials; (3) diagnostically ambiguous or complex cases precluding definitive conclusions; (4) termination of the appraisal process; and (5) cases deemed professionally unsuitable for SSDC assessment.

### 2.2 Research methods

#### 2.2.1 SSDC assessment criteria

Three senior forensic examiners conducted the SSDC assessments according to the Guidelines for Assessment of SSDC in the Mentally Disordered (SF/T 0071-2020), concurrently administering the Capacity of Sexual Self-defense Assessment Scale (CSSAS). The CSSAS is a nationally mandated standardized tool in Chinese forensic psychiatry for assessing sexual self-defense capacity (SSDC). This 39-point scale evaluates cognitive, behavioral, emotional, and social support dimensions, with higher scores indicating better SSDC. Its three scoring tiers directly correspond to statutory SSDC assessment levels. Developed through expert consensus, the scale aligns with China’s Criminal Law and SSDC assessment guidelines, ensuring content validity. Criterion validity is established through consistent alignment with judicial rulings, while mandatory centralized training and certification ensure inter-rater reliability. This 39-point scale classifies SSDC as Absent (CSSAS Score < 7), Diminished (7 ≤ CSSAS Score < 21), or Retained (CSSAS Score ≥ 21), with CSSAS results required to align with the overall SSDC determination (23).

Qualified psychometric professionals administered the China-revised Wechsler Adult/Children Intelligence Scales and the Activities of Daily Living Scale (ADL) to evaluate Intellectual Level and ADL, respectively. The China-revised Wechsler Adult/Children Intelligence Scales and the Activities of Daily Living Scale (ADL) are well-established instruments with demonstrated good reliability and validity in Chinese populations, as cited in our references (25, 24–27). Electroencephalogram (EEG) and cerebral Computed Tomography (CT) scans were performed to detect abnormalities, while physical disability status was assessed using the WHO International Classification of Functioning, Disability and Health (ICF) framework. CSSAS Score, representing the level of SSDC, was analyzed in relation to: general characteristics (age, education level, employment status, marital status); sexual behavior history (previous sexual behavior, pregnancy history, pregnancy resulting from the alleged sexual offense); medical findings (EEG results, CT results, physical disability status, mental disorder diagnosis); and social functioning (intellectual level, ADL) (28, 29).

#### 2.2.2 Definition of specialized terms

Previous Sexual Behavior History was categorized based on information provided by the commissioning party, the examinee, and their relatives, as well as relevant medical examinations. Previous sexual behavior and pregnancy history were defined as the examinee’s sexual and obstetric history prior to the alleged incident, while pregnancy resulting from the alleged sexual offense specifically referred to pregnancies caused by the reported assault.

Medical diagnosis was classified according to ICD-10 and CCMD-3 into three groups: Intellectual disability, Schizophrenia, and other mental disorders. The grouping criteria prioritized sample size, with less prevalent conditions—such as organic mental disorders, mood disorders, unspecified non-organic psychoses, neurotic disorders, and substance-induced mental disorders—consolidated into the other mental disorders category due to insufficient case numbers.

### 2.3 Statistical analysis

Statistical analysis was performed using SPSS 26.0. Continuous variables were expressed as mean ± standard deviation ( $\bar{x}$ ± s). Independent samples t-test was used for comparisons between two groups, while one-way ANOVA (F-test) was applied for multi-group comparisons, followed by the Student-Newman-Keuls (SNK-q) *post hoc* test for pairwise analysis. Pearson correlation analysis was conducted to examine the relationship between CSSAS scores and numerical variables. A multiple linear regression model was constructed to assess predictive factors, and receiver operating characteristic (ROC) curve analysis was performed to evaluate the diagnostic efficacy of Intellectual Level (FSIQ) and Activities of Daily Living (ADL) scores. Binary logistic regression was used to calculate the combined predictive probability of ADL and FSIQ on SSDC, and Z-test was employed to compare the area under the curve (AUC) values among different models. A two-tailed P-value < 0.05 was considered statistically significant.

## 3 Results

### 3.1 General characteristics

The study analyzed 398 female examinees undergoing SSDC assessments, showing: (1) mean age 33.86 ± 15.29 years; (2) illiteracy prevalence at 52.76%; (3) unemployed status in 83.67%; (4) with marital history (55.28%); (5) with pregnancy history (50.75%); (6) current pregnancy status negative (92.71%); (7) with previous sexual behavior (56.53%); (8) normal EEG results (81.41%); (9) normal CT results (89.45%); (10) absence of physical disabilities (94.22%); (11) Intellectual disability as primary diagnosis (65.08%); with cognitive and functional assessments revealing (12) FSIQ 57.21 ± 18.42, VIQ 56.97 ± 18.81, PIQ 56.33 ± 18.93; and (13) ADL Score 32.61 ± 9.74.

### 3.2 Univariate analysis of SSDC assessment results and influencing factors

The univariate analysis of categorical variables revealed statistically significant differences (P<0.05) in CSSAS Scores across several factors: (1) Education level showed illiteracy group scoring significantly lower than primary school and junior high school or above groups; (2) Employment Status demonstrated employed examinees having higher CSSAS Scores than unemployed counterparts; (3) Current Pregnancy Status indicated non-pregnant cases scored higher than pregnancy-resulting-from-offense cases; (4) EEG Results displayed abnormal EEG group scoring lower than normal EEG group; (5) CT Results showed abnormal CT group with lower scores than normal CT group; (6) Medical Diagnosis revealed intellectual disability group scoring significantly lower than schizophrenia and other mental disorder groups, with an increasing score trend observed. No statistically significant differences (P>0.05) were found for marital status, pregnancy history, prior sexual behavior, or physical disability status (see **Table 1**).

**Table 1:** Single factor analysis of CSSAS score of 398 cases and related influencing factors.

Group	Subgroup (n)	CSSAS score (MD ± SD)	*t/F*	*P*
Education level	Illiteracy (*n* = 210)	7.31 ± 6.92_a_	62.70	0.00
	Primary school (*n* = 86)	13.35 ± 9.69_b_		
	Junior high school or above (*n* = 102)	19.31 ± 11.81_c_		
Employment Status	Unemployed (*n* = 333)	10.10 ± 9.47	55.34	0.00
	Employed (*n* = 65)	19.86 ± 10.69		
Marital Status	No marital history (*n* = 178)	12.66 ± 11.11	2.86	0.09
	With marital history (*n* = 220)	10.91 ± 9.58		
Pregnancy History	No pregnancy history (*n* = 196)	12.36 ± 11.01	1.60	0.21
	With pregnancy history (*n* = 202)	11.05 ± 9.59		
Current Pregnancy Status	Not pregnant (*n* = 369)	11.7 ± 10.469	2.68	0.01
	Pregnant (*n* = 29)	7.21 ± 8.541		
Previous Sexual Behavior History	No prior sexual behavior (*n* = 173)	12.20 ± 10.74	0.73	0.39
	With prior sexual behavior (*n* = 225)	11.31 ± 9.99		
EEG Results	Normal (*n* = 324)	12.28 ± 10.53	5.71	0.02
	Abnormal (*n* = 74)	9.12 ± 8.96		
CT Results	Normal (*n* = 356)	12.06 ± 10.56	4.34	0.04
	Abnormal (*n* = 42)	8.57 ± 7.40		
Physical Disability Status	No physical disability (*n* = 375)	11.85 ± 10.46	1.51	0.22
	With physical disability (*n* = 23)	9.13 ± 7.48		
Medical Diagnosis	Intellectual disability (*n* = 259)	7.87 ± 6.75_a_	122.00	0.00
	Schizophrenia (*n* = 67)	12.40 ± 8.40_b_		
	Others (*n* = 72)	24.78 ± 11.63_c_		

SNK-q test for multiple comparisons. Letters (a, b, c) indicate homogeneous subsets (α=0.05) when compared with the multi-site group.

### 3.3 Correlation analysis between SSDC scores and related influencing factors

Pearson correlation analysis of numerical variables revealed the following key findings: CSSAS Scores showed a strong positive correlation with Intellectual Level (FSIQ: r = 0.860, VIQ: r = 0.827, PIQ: r = 0.836, all p< 0.001), confirming Intellectual Level as a critical determinant of SSDC. Due to the high multicollinearity between FSIQ, VIQ, and PIQ (with FSIQ encompassing multiple cognitive domains), further analysis focused solely on FSIQ as the representative measure of Intellectual Level. CSSAS Scores demonstrated a strong negative correlation with ADL Scores (r = -0.861, p< 0.001), indicating that worse daily living functioning was associated with weaker SSDC.A weak but significant negative correlation existed between CSSAS Scores and Age (r = -0.170, p = 0.001), suggesting Age had a limited influence on SSDC (see **Table 2**).

**Table 2:** Correlation analysis between CSSAS scores and associated influencing factors in 398 cases.

Dependent variable / Independent variable		Age	Intellectual level			ADL Score
			FSIQ	VIQ	PIQ	
CSSAS Score	*r*	-0.170**	0.860**	0.827**	0.830**	-0.861**
	*p*	0.001	0.000	0.000	0.000	0.000

### 3.4 Multiple linear regression analysis of factors influencing SSDC scores

The correlation analysis demonstrated statistically significant associations (P < 0.05) between CSSAS Scores and education level, employment status, current pregnancy status, EEG results, CT results, and medical diagnosis. CSSAS Scores showed a positive correlation with Intellectual Level (with FSIQ selected as the sole representative measure due to strong multicollinearity among FSIQ, VIQ and PIQ), while exhibiting strong negative correlations with ADL scores and age.

Consequently, in the multiple linear regression analysis examining factors influencing CSSAS Scores (using CSSAS Scores as the dependent variable and age, education level, employment status, current pregnancy status, EEG results, CT results, medical diagnosis, FSIQ, and ADL scores as independent variables, with dummy variables set for education level using the illiteracy group as reference and for medical diagnosis using the other disorders group as reference), the standardized regression coefficients (β) revealed the following order of influence magnitude: ADL scores (β = -0.409), FSIQ (β = 0.379), schizophrenia diagnosis (β = -0.199), intellectual disability diagnosis (β = -0.157), and employment status (β = 0.116).

The regression results demonstrated several significant findings: For each 1-point increase in ADL scores (indicating worse daily functioning), CSSAS Scores decreased by 0.436 points (P<0.001), confirming daily living ability as the strongest predictor. A 1-point improvement in FSIQ was associated with a 0.214-point increase in CSSAS Scores (P<0.001). Employed individuals showed 3.17-point higher CSSAS Scores than unemployed counterparts (P<0.001). Analysis of categorical variables revealed that, compared to other mental disorders, both intellectual disability (B=-3.413, β=-0.157, P<0.001) and schizophrenia (B=-5.519, β=-0.199, P<0.001) were independent risk factors for impaired SSDC, with schizophrenia exhibiting a greater negative effect (β=-0.199 vs -0.157). The 95% confidence intervals for all significant variables excluded zero, confirming result reliability. Regarding education level (reference: illiteracy), neither primary school (B=-0.051, P = 0.941) nor junior high school and above (B = 0.202, P = 0.790) showed statistically significant effects. Notably, EEG results, CT findings, age, and current pregnancy status also demonstrated no significant associations (P>0.05) (**Table 3**).

**Table 3:** Multiple linear regression analysis of factors associated with CSSAS scores in 398 cases.

Independent variable		Unstandardized coefficients		Standardized coefficients	95% CI for B		*t*	*P*
		B	SE	β	Lower bound	Upper bound		
Employment status (Unemployed=1)		3.261	0.670	0.116	1.943	4.579	4.865	0.000
EEG Results (Normal=1)		-1.081	0.702	-0.040	-2.460	0.299	-1.540	0.124
CT Results (Normal=1)		0.134	0.862	0.004	-1.561	1.829	0.155	0.877
FSIQ		0.214	0.040	0.379	0.136	0.292	5.377	0.000
ADL score		-0.436	0.067	-0.409	-0.569	-0.304	-6.483	0.000
Age		0.002	0.019	0.003	-0.035	0.039	0.109	0.913
Current Pregnancy Status (Not pregnant=1)		0.009	0.936	0.000	-1.831	1.848	0.009	0.993
Education level	Primary school	-0.051	0.685	-0.002	-1.398	1.296	-0.074	0.941
	Junior high school or above	0.202	0.759	0.008	-1.289	1.693	0.266	0.790
Medical Diagnosis	Intellectual disability	-3.413	0.916	-0.157	-5.213	-1.612	-3.727	0.000
	Schizophrenia	-5.519	0.892	-0.199	-7.272	-3.766	-6.190	0.000

B, Regression coefficient; SE, Standard error; CI, Confidence interval; β, Standardized regression coefficient.

The model exhibited strong explanatory power (R²=0.801, adjusted R²=0.795), accounting for 79.5% of variance in CSSAS Scores, with a standard error of estimate of 4.704 indicating high predictive accuracy (**Table 4**).

**Table 4:** Goodness-of-fit indicators for the multiple linear regression model of factors influencing CSSAS scores.

Model summary			
R	R^2^	Adjusted R^2^	Std. error of the estimate
.895^a^	.801	.795	4.704

^a^Predictors: (Constant), Employment status, EEG Results, CT results, FSIQ, ADL score, Age, Current Pregnancy Status, Primary school, Junior high school or above, Schizophrenia, intellectual disability.

### 3.5 ROC curve analysis of factors influencing sexual self-defense capacity

The ROC curve analysis evaluated the diagnostic performance of ADL scores and FSIQ in predicting SSDC impairment at two thresholds: CSSAS Scores <21 and CSSAS Scores <7.

**Figure 1:**
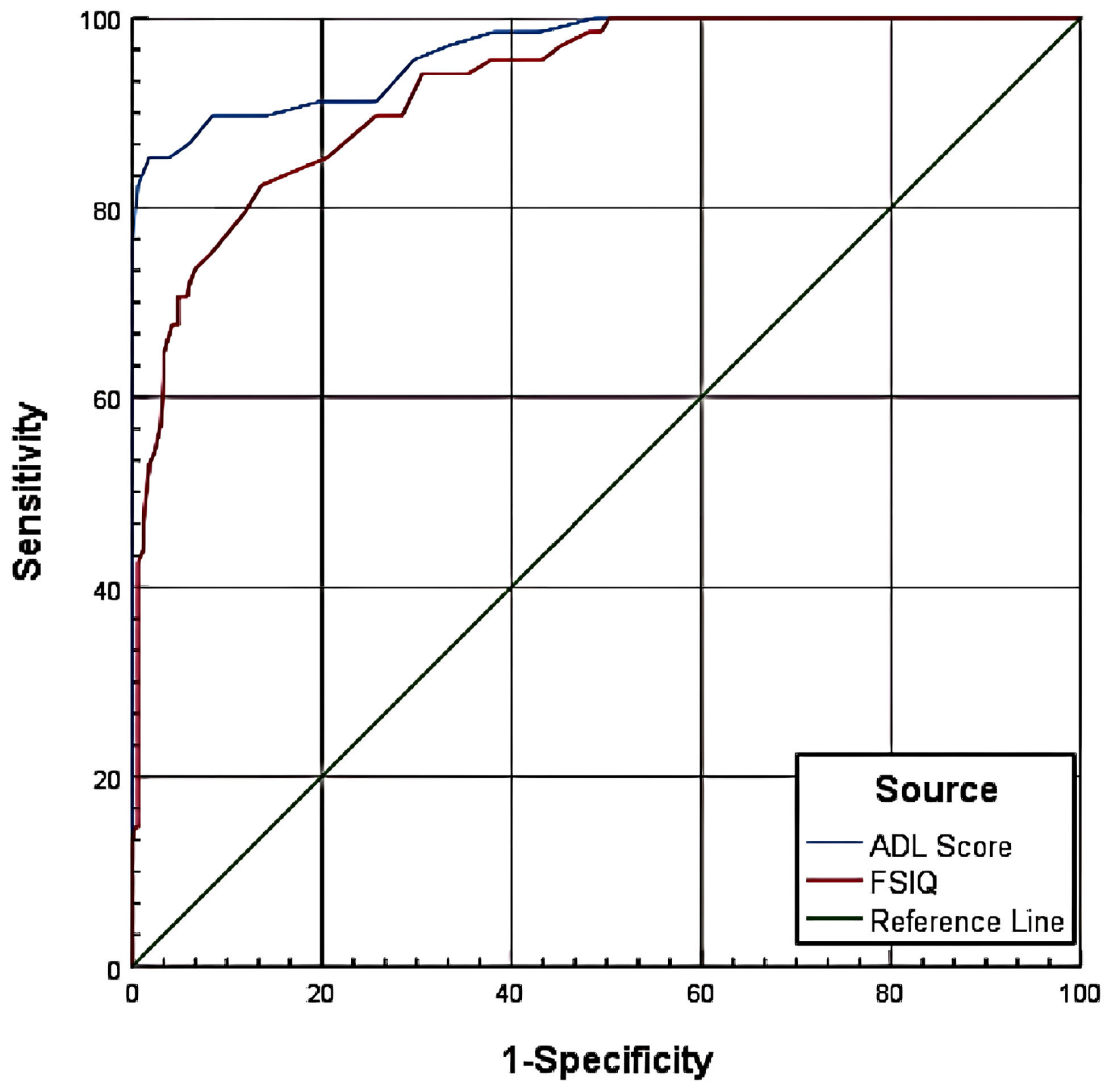
ROC curve for CSSAS scores <21.

**Figure 2:**
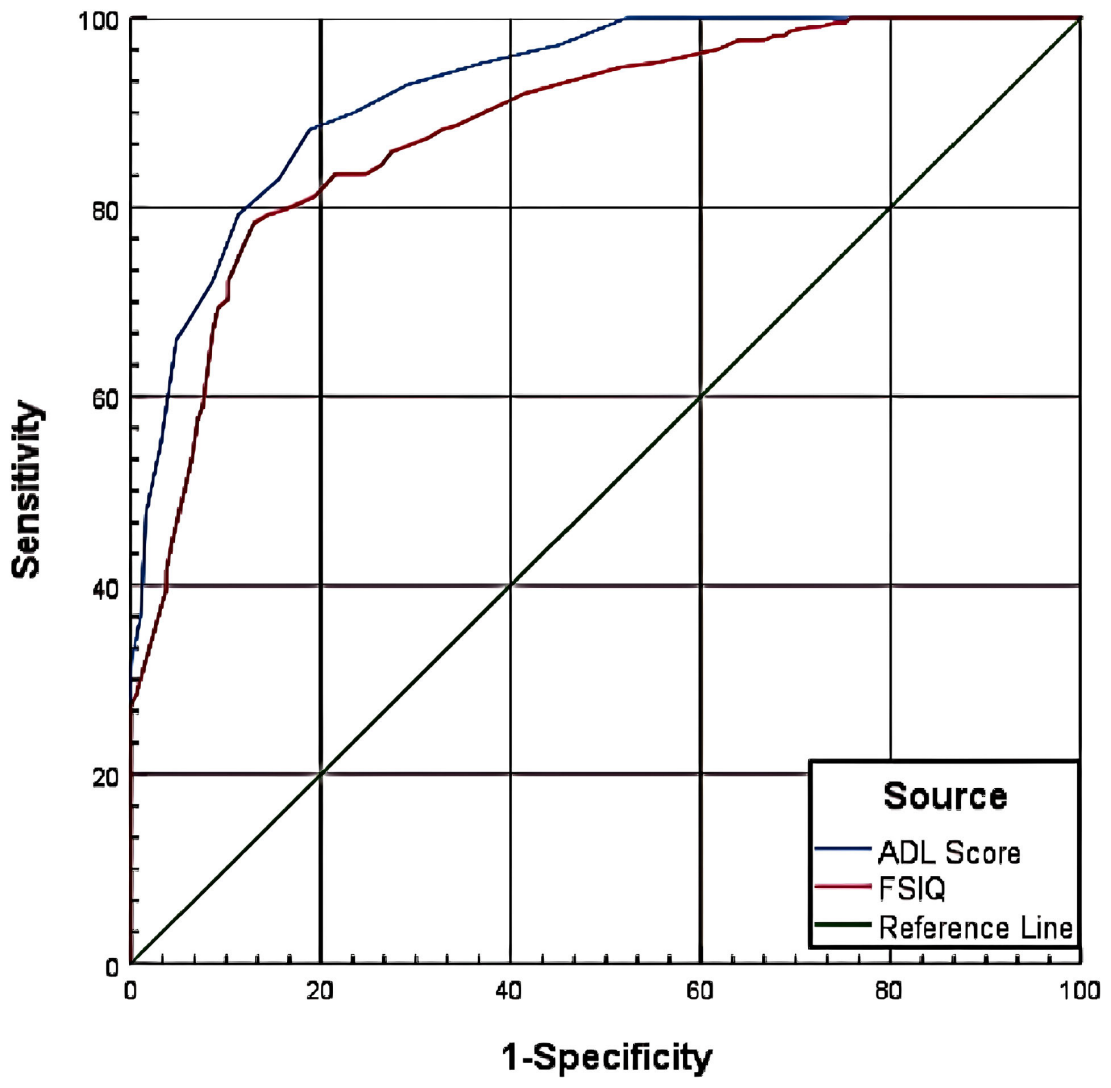
ROC curve for CSSAS scores <7.

For CSSAS Scores <21, ADL scores yielded an AUC of 0.965, with optimal diagnostic cutoff at 22.5 (specificity=85.30%, sensitivity=98.20%), while FSIQ showed an AUC of 0.925 with optimal cutoff at 66.5 (specificity=86.40%, sensitivity=82.40%). For CSSAS Scores <7, ADL scores demonstrated an AUC of 0.926 with cutoff at 31.5 (specificity=81.20%, sensitivity=88.20%), and FSIQ achieved an AUC of 0.885 with cutoff at 53.5 (specificity=87.10%, sensitivity=78.30%). The results indicate that both ADL scores and FSIQ have significant diagnostic value for assessing impaired sexual self-defense capacity, with ADL scores showing superior performance in terms of AUC values and sensitivity at both cutoff thresholds (**Table 5**; **Figures 1**, **2**).

**Table 5:** Diagnostic model for CSSAS scores.

Variable	AUC	SE	95%CI	*P* value	Cutoff value	Specificity	Sensitivity
CSSAS score <21							
FSIQ	0.925	0.016	0.894-0.957	<0.0001	66.5	86.40%	82.40%
ADL Score	0.965	0.012	0.942-0.989	<0.0001	22.5	85.30%	98.20%
CSSAS score <7							
FSIQ	0.885	0.016	0.853 - 0.917	<0.0001	53.5	87.10%	78.30%
ADL Score	0.926	0.029	0.902- 0.950	<0.0001	31.5	81.20%	88.20%

AUC: Area under the curve; SE: Standard error; CI: Confidence interval; Cutoff Value: Threshold value.

### 3.6 Combined predictive value of ADL scores and FSIQ for sexual self-defense capacity

#### 3.6.1 Combined prediction for CSSAS scores <7

**Figure 3:**
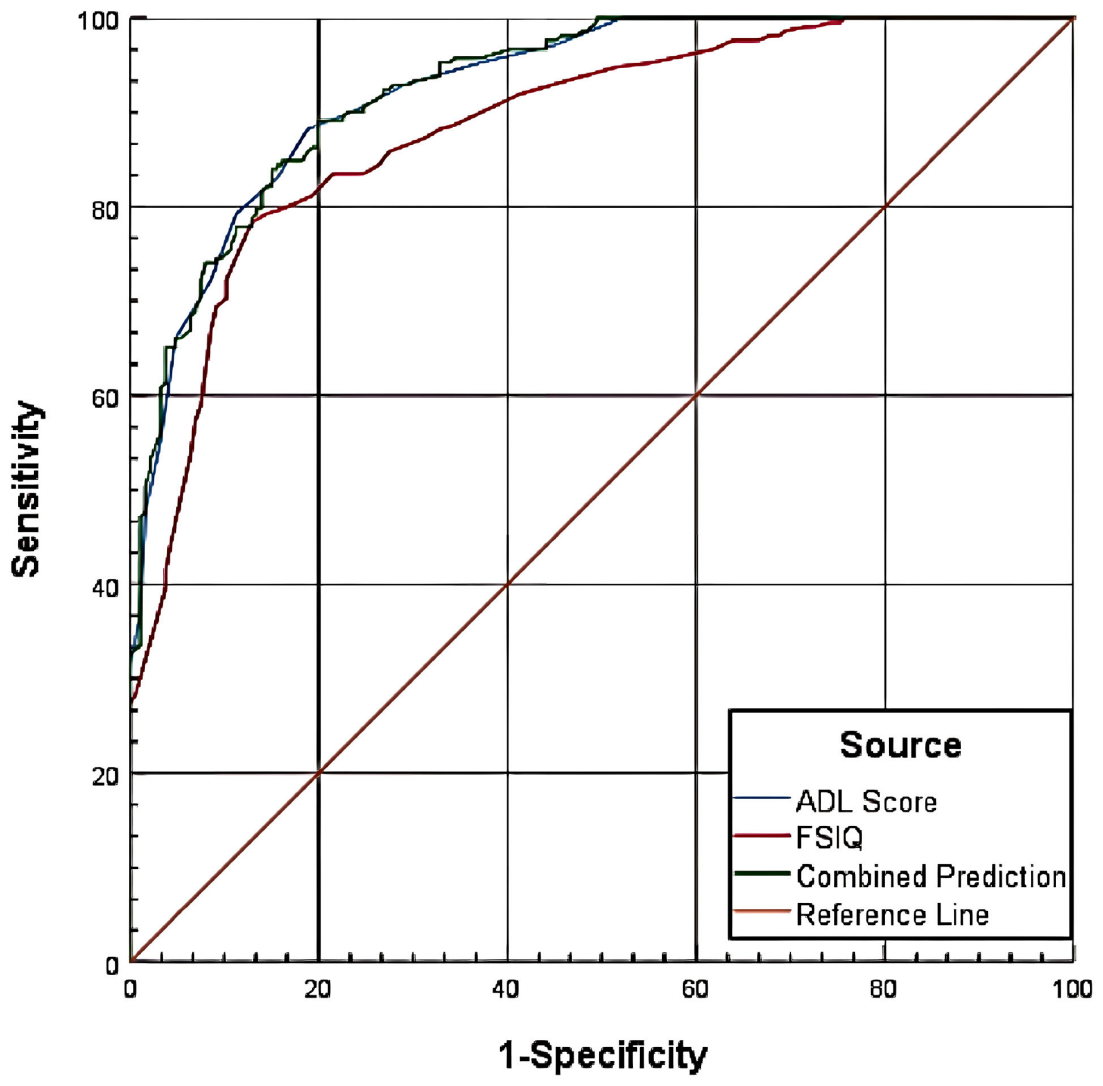
Combined predictive model for CSSAS scores <7.

The combined prediction model incorporating both ADL scores and FSIQ demonstrated superior performance (AUC = 0.927) compared to either measure alone (P<0.05). Pairwise comparisons revealed three key findings: First, ADL scores (AUC = 0.926) showed significantly greater predictive accuracy than FSIQ (AUC = 0.885), with a difference of 0.0411 (Z = 4.409, P<0.0001), indicating ADL’s superior discriminative ability between positive and negative cases. Second, the combined model’s performance (AUC = 0.927) did not significantly improve upon ADL scores alone (difference=0.001, Z = 0.589, P = 0.5557). Third, the combined model outperformed FSIQ alone (difference=0.0421, Z = 3.925, P = 0.0001), confirming enhanced classification accuracy when combining both measures. These results demonstrate that while ADL scores alone provide robust prediction, the combination with FSIQ offers meaningful improvement over using FSIQ in isolation (**Table 6**, **Figure 3**).

**Table 6:** Diagnostic performance of ADL scores and FSIQ for CSSAS scores <7.

Variable	AUC	SE	*P* value	Asymptotic 95% CI	
				Lower bound	Upper bound
ADL Score	0.926	0.012	0.000	0.902	0.950
FSIQ	0.885	0.016	0.000	0.853	0.917
Combined Prediction	0.927	0.012	0.000	0.903	0.951

#### 3.6.2 Combined prediction for CSSAS scores <21

**Figure 4:**
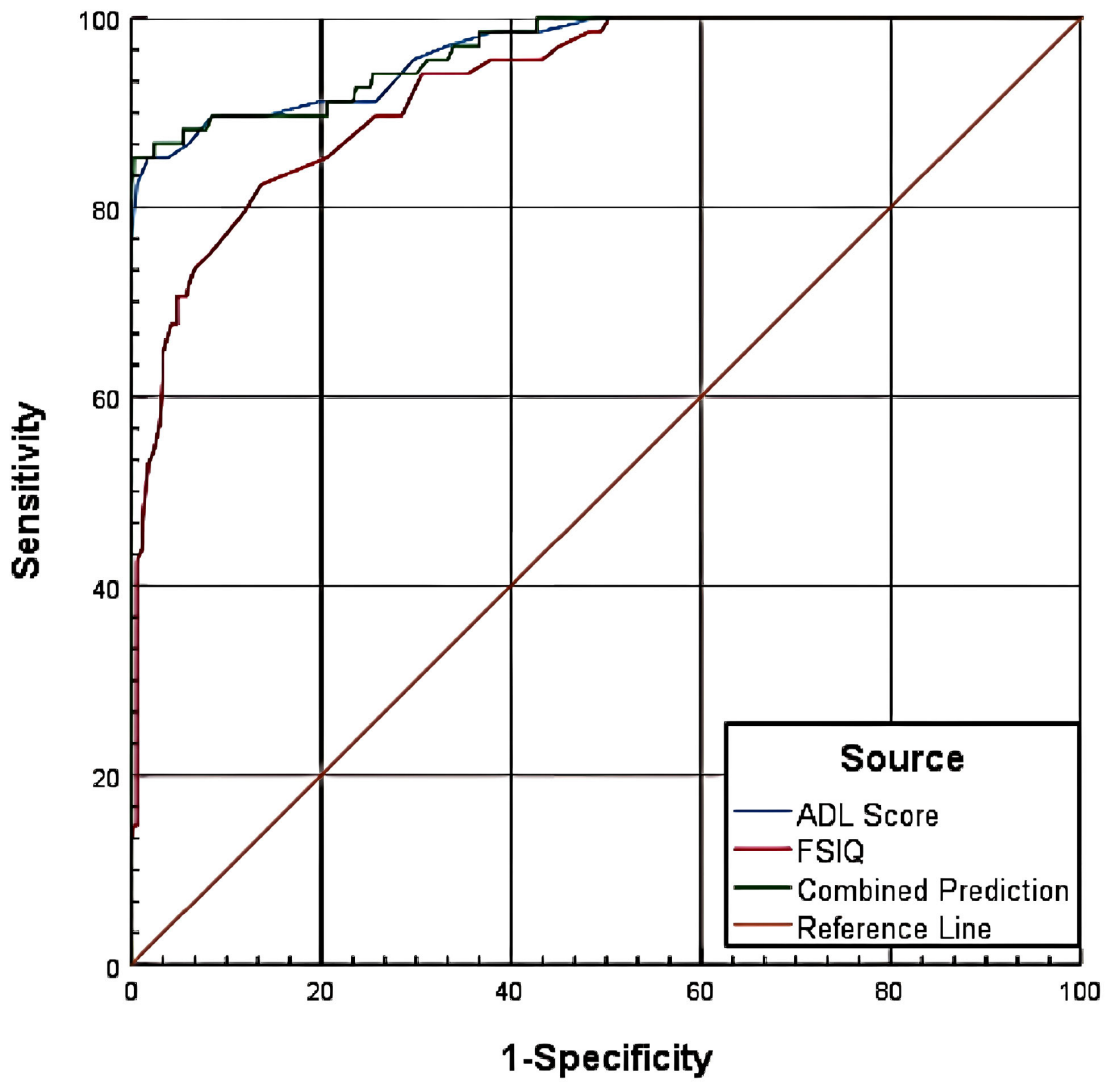
Combined predictive model for CSSAS scores <21.

The combined predictive model integrating ADL scores and FSIQ achieved an AUC of 0.966 for identifying CSSAS Scores <21, significantly outperforming individual measures (P<0.05). Comparative analysis revealed three principal findings: First, ADL scores (AUC = 0.955) demonstrated significantly greater discriminative power than FSIQ (AUC = 0.925), with a difference of 0.0398 (Z = 3.721, P = 0.0002), confirming ADL’s superior performance in classification. Second, the combined model showed no significant improvement over ADL scores alone (difference=0.011, Z = 0.481, P = 0.6302). Third, the combined approach provided substantially better prediction than FSIQ in isolation (difference=0.0408, Z = 3.374, P = 0.0007). These results collectively indicate that while the combined model enhances predictive accuracy relative to FSIQ alone, ADL scores remain the dominant predictor for this threshold, with minimal incremental value from incorporating FSIQ (**Table 7**, **Figure 4**).

**Table 7:** Diagnostic performance of ADL scores and FSIQ for CSSAS scores <21.

Variable	AUC	SE	*P* value	Asymptotic 95% CI	
				Lower bound	Upper bound
ADL Score	0.965	0.012	0.000	0.942	0.989
FSIQ	0.925	0.016	0.000	0.894	0.957
Combined Prediction	0.966	0.012	0.000	0.943	0.990

## 4 Discussion

The assessment of sexual self-defense capacity, as a core issue in forensic psychiatric practice, presents three major limitations in current evaluation systems regarding scientific rigor and objectivity. First, the singular focus on assessment indicators predominates international research, with excessive reliance on intelligence testing (e.g., WAIS scale) or psychiatric symptom evaluation while neglecting the behavioral dimension of daily living functioning (18). Second, the diagnostic criteria demonstrate problematic ambiguity - although China’s Guidelines for Assessment of Sexual Self-defense Capacity in the Mentally Disordered proposes a three-tier classification framework, it lacks objective cutoff values, leading to inconsistent evaluation outcomes in practice (8, 9). Third, forensic assessments frequently demonstrate incomplete consideration of examinees’ comprehensive information, overemphasizing specific indicators (e.g., intelligence level, medical examination results) while overlooking fundamental characteristics (e.g., employment history, sexual behavior history). These methodological deficiencies have contributed to divergent expert opinions in judicial practice (20).

To address these limitations, the current study analyzed 398 forensic evaluation cases to systematically examine factors influencing sexual self-defense capacity in mentally disordered individuals and develop a predictive model. This research represents a methodological advance beyond the traditional trichotomous classification (“present/absent/partial”) by employing quantitative scale scores to precisely capture the continuous spectrum of sexual self-defense capacity. Through ROC curve analysis, we established optimal cutoff values with maximum sensitivity and specificity, thereby deriving objective quantitative standards for ADL Scores and FSIQ in capacity assessments - a significant improvement over previous subjective judgment-based approaches. Furthermore, our model incorporated both basic demographic information and neurophysiological indicators (EEG, CT scans), effectively compensating for the shortcomings of purely psychological evaluations. Most importantly, we developed and validated a combined assessment model integrating ADL and FSIQ, empirically demonstrating the necessity of multidimensional evaluation in forensic practice.

### 4.1 Analysis of general demographic characteristics

The analysis revealed significant associations between demographic factors and CSSAS Scores (p<0.001). Education level demonstrated a particularly strong influence, with illiterate individuals showing significantly lower CSSAS Scores compared to those with junior high school education or above. This finding carries important implications for legal determinations in rape cases, as education may enhance cognitive and behavioral capacities relevant to recognizing and responding to sexual threats. Notably, the high proportion of illiterate individuals in our sample (52.76%) suggests that limited education may represent a significant risk factor for impaired sexual self-defense capacity, highlighting the protective value of educational attainment (30).

Employment status similarly showed significant associations (p<0.001), with unemployed individuals exhibiting lower CSSAS Scores than their employed counterparts. Multiple linear regression confirmed employment as an independent predictor of sexual self-defense capacity, potentially because employment fosters protective factors through enhanced social interaction, improved situational judgment, greater economic independence (reducing vulnerability to coercion), and expanded social support networks (31). The predominance of unemployed individuals in our sample (83.67%) may reflect the broader challenges of workforce participation among those with mental disorders due to impaired social functioning.

Current pregnancy status following the alleged assault also showed significant differences (p=0.011), with pregnancy resulting from the offense associated with lower CSSAS Scores. This pattern suggests that individuals with impaired sexual self-defense capacity may demonstrate reduced ability to protect themselves during assaults, potentially increasing their vulnerability to more severe consequences including pregnancy. These findings collectively underscore the importance of considering socioeconomic and situational factors in sexual self-defense capacity assessments.

Furthermore, correlation analysis revealed that age, while showing a relatively weak association (p<0.05), nevertheless demonstrated statistical significance. This finding reflects the current status of sexual self-defense capacity among extreme age groups. On one hand, adolescents’ incomplete neurocognitive development may contribute to inadequate sexual self-defense capacity. Studies indicate that adolescents face higher risks of sexual violence compared to other age groups (32, 33), with intellectually disabled adolescents being particularly vulnerable (34). On the other hand, natural cognitive decline in elderly populations may impair risk assessment abilities (35). These age-related differences suggest the need to incorporate developmental factors into assessment criteria.

However, no significant differences in CSSAS Scores were observed for marital status, pregnancy history, prior sexual behavior, or physical disability status (p=0.092, 0.21, 0.39, 0.22 respectively). This may be because factors like marital status, pregnancy history, and sexual behavior are more closely related to sexual knowledge rather than directly reflecting sexual self-defense capacity. For instance, some married individuals or those with pregnancy history may have acquired sexual knowledge passively without developing a comprehensive cognitive framework for sexual self-protection. Additionally, our evaluation focused specifically on cognitive aspects of sexual self-defense capacity (36) - the ability to comprehend sexual situations. While physical disabilities may impair social functioning, they typically do not directly affect these cognitive capacities unless causing concomitant intellectual impairment.

### 4.2 Analysis of neuropsychological indicators

The neurophysiological examination revealed significantly lower CSSAS Scores in individuals with abnormal EEG and CT findings (p<0.05), providing objective evidence for the association between neural integrity and sexual cognition. These results suggest the need to establish standardized protocols for incorporating neuroimaging data as essential components of SSDC assessments (37).

Medical diagnosis significantly influenced sexual self-defense capacity, with our findings consistent with prior research showing higher prevalence of intellectual disability and schizophrenia among forensic cases with impaired SSDC (38, 39). Specifically, the intellectual disability group demonstrated significantly lower CSSAS Scores than both schizophrenia and other diagnostic groups, indicating distinct pathological mechanisms. Intellectual disability primarily compromises fundamental cognitive capacities - particularly the ability to recognize and evaluate risky sexual situations - suggesting that basic cognitive impairment may more substantially impact SSDC than psychotic symptoms, as supported by Nixon’s (2017) work (35). In contrast, schizophrenia may additionally impair SSDC through symptom-driven limitations (e.g., delusions, thought disorder) that restrict help-seeking and self-protective behaviors (40).

Analysis of cognitive and functional measures yielded two key findings: First, intellectual level showed strong positive correlations with CSSAS Scores (FSIQ, VIQ, PIQ all p<0.001), confirming its central role in SSDC assessments. The slightly stronger correlation for PIQ versus VIQ suggests behavioral responsiveness and spatial judgment may be more critical than verbal comprehension in actual assault scenarios. Second, ADL Scores demonstrated a robust negative correlation with CSSAS Scores (p<0.001), with research indicating daily functioning deficits may impair both threat recognition and physical capacity to escape dangerous situations (41). Regression analysis identified FSIQ (β=0.379) and ADL Scores (β=-0.409) as the strongest predictors (both p<0.001), with ADL’s marginally greater absolute β-value suggesting practical daily functioning may be more informative than intellectual scores alone - a finding subsequently corroborated by ROC analysis.

### 4.3 Predictive model for sexual self-defense capacity assessment

This study established objective criteria for ADL Scores and FSIQ in sexual self-defense capacity (SSDC) assessments through ROC analysis, with findings of significant value for rape case adjudication and criminal justice practice. Based on the Guidelines for Assessment of Sexual Self-defense Capacity in the Mentally Disordered and CSSAS Scores, we developed a three-tiered SSDC classification system: when ADL ≤ 22.5 or FSIQ≥66.5, CSSAS Scores≥21 indicate preserved SSDC; when 22.5<ADL ≤ 31.5 or 53.5≤FSIQ<66.5, 7≤CSSAS Scores<21 indicate diminished SSDC; and when ADL>31.5 or FSIQ<53.5, CSSAS Scores<7 indicate absent SSDC. Caution is warranted when applying these cutoffs: first, cultural and social environmental factors may influence cognitive or behavioral performance, potentially requiring standard adjustments in cross-cultural applications (42); second, comprehensive multidimensional assessment is recommended for borderline cases (e.g., FSIQ:60–70 or ADL:20-25) to enhance accuracy; third, developmental or degenerative factors require special consideration when applying these standards to special populations like adolescents or the elderly. These findings not only support the central role of cognitive-behavioral functioning in SSDC assessment but also provide a scientifically grounded and operationally feasible framework for forensic practice.

Combined prediction analysis demonstrated that when “CSSAS Scores<7”, ADL’s AUC (0.926) was significantly higher than FSIQ’s (0.885), with a difference of 0.0411, indicating ADL’s superior predictive ability in distinguishing between absent SSDC versus preserved/diminished SSDC groups. Moreover, the combined model’s AUC was significantly higher than FSIQ alone (difference=0.0421), confirming better assessment efficacy. Similarly, when “CSSAS Scores<21”, ADL’s AUC (0.955) significantly outperformed FSIQ (0.925) by 0.0398, showing ADL’s greater discriminative power between preserved versus absent/diminished SSDC groups. The combined model’s AUC was also significantly higher than FSIQ alone (difference=0.0408), demonstrating improved classification accuracy.

However, the study found that for both “CSSAS Scores<7” and “CSSAS Scores<21”, ADL and combined prediction AUC values were nearly identical with non-significant differences, suggesting the combined model did not substantially improve upon ADL’s predictive performance. This likely stems from ADL Scores already encompassing core elements of cognitive functioning like intelligence (43), achieving near-maximal predictive efficacy (AUC>0.92) that leaves little room for improvement. This finding carries important clinical implications: ADL should be prioritized as a screening tool in SSDC assessments while still incorporating FSIQ, maintaining accuracy while significantly improving forensic evaluation efficiency - particularly valuable for resource-limited basic forensic institutions. These results align with WHO’s International Classification of Functioning (ICF) concept that “daily living functioning integrates cognitive and social adaptation” (29), providing empirical support for streamlining forensic psychiatric evaluations while validating the scientific and practical utility of the three-tiered classification system.

The three-tiered criteria hold significant guidance value for judicial practice in rape cases. During criminal investigations, these standards help investigators preliminarily identify victims’ SSDC status: for victims with ADL>31.5 and FSIQ<53.5 (absent SSDC), investigators should prioritize evidence proving the perpetrator’s “knowledge” of the victim’s special status; for victims with diminished SSDC, assessments should integrate cognitive status with case specifics. In forensic evaluations, these standards provide objective criteria for determining whether victims possess the “capacity to recognize and uphold their right to sexual inviolability” - a key element in rape cases, where “consent” is legally invalid for victims deemed to lack SSDC. For sentencing, China’s criminal law mandates harsher punishment for sexual offenses against individuals known to lack SSDC (44), and this study’s operational criteria for establishing “knowledge” help courts achieve proportional sentencing. This classification system will enhance the scientific rigor and accuracy of sexual assault case handling, avoiding revictimization of vulnerable groups while ensuring judicial fairness.

In summary, ADL Scores and FSIQ provide more comprehensive and precise diagnostic tools for SSDC assessment. Both measures demonstrate good diagnostic value across different classification standards, with combined use offering modest predictive improvement. In all analyses, ADL Scores outperformed FSIQ, a finding with important theoretical and practical implications. Theoretically, this occurs because ADL Scores encompass not only cognitive components but also more comprehensive dimensions including cognitive and behavioral capacities (43). Ultimately, these results confirm the primary value of daily living functioning assessments in identifying defense capacity deficits, particularly for screening vulnerable populations needing protection, where ADL’s comprehensive evaluation capacity ensures minimal oversight of high-risk individuals, while FSIQ provides valuable complementary specificity that enhances result reliability, further validating the necessity of multidimensional assessment (45).

### 4.4 Conclusions

This study systematically analyzed multi-dimensional factors such as general information, medical diagnosis, and neurophysiological indicators, identified the key predictors affecting SSDC, and established an objective and standardized assessment system. When ADL score ≤22.5 or FSIQ ≥ 66.5, CSSAS score ≥ 21 indicates retained SSDC. When 22.5 < ADL score ≤ 31.5 or 53.5 ≤ FSIQ < 66.5, CSSAS score between 7 and 21 (inclusive of 7, exclusive of 21) indicates diminished SSDC. When ADL score > 31.5 or FSIQ < 53.5, CSSAS score < 7 indicates the absence of SSDC. These numerical criteria are based on large-sample data analysis and hold significant practical value in SSDC identification. Specifically:

First, at the level of forensic identification, the ADL score can effectively identify deficits in the evaluated individual’s ability to function in daily life scenarios, while the FSIQ reflects, from the perspective of cognitive function, the individual’s ability to recognize and respond to sexual assault. The combined application of the two not only significantly enhances the scientific validity and reliability of forensic conclusions, but also provides crucial evidential support for judicial authorities in determining the facts of rape cases; through multi-dimensional quantitative indicators, this joint assessment model enables the establishment of a more comprehensive and objective system for evaluating SSDC.

Second, during the criminal investigation phase, law enforcement agencies can conduct a scientific screening of the SSDC status of individuals involved in the case at the early stage, based on the standardized assessment results of ADL and FSIQ. This screening not only helps investigators quickly determine the nature of the case, but also guides them in formulating targeted investigation plans. For instance, if the preliminary assessment shows that the involved individual has significant ADL functional deficits or a low FSIQ level, the law enforcement agency should promptly initiate formal forensic identification procedures, while paying attention to adopting special questioning methods to ensure the legality of the evidence collection process and the validity of the evidence. This measure not only optimizes the allocation of judicial resources, but also effectively avoids delays in the case-handling process caused by delayed assessments.

Furthermore, during sentencing, the quantitative results of ADL and FSIQ derived from this assessment system can serve as important reference bases for courts to determine penalties. On one hand, if the assessment clearly shows that the victim has absent SSDC due to ADL score > 31.5 or FSIQ < 53.5, or has diminished SSDC due to an ADL score between 22.5 and 31.5 (exclusive of 22.5, inclusive of 31.5) or an FSIQ between 53.5 and 66.5 (inclusive of 53.5, exclusive of 66.5), the court usually identifies this circumstance as an aggravating factor for punishment. Within the statutory sentencing range for rape (such as fixed-term imprisonment or life imprisonment), the court will appropriately increase the term of imprisonment for the defendant, taking into account case details like the means of the crime and the harmful consequences. On the other hand, clear quantitative standards can reduce sentencing discrepancies caused by ambiguous identification of “SSDC” in judicial practice, ensuring more consistency and fairness in the punishment of similar cases. This not only fully protects the victim’s rights and interests through reasonable sentencing, but also exerts the deterrent effect of criminal penalties in preventing such crimes through a clear punishment scale. However, it must be unequivocally stated that the results derived solely from this system are insufficient to serve as a basis for sentencing and function only as a reference. The final judicial ruling must integrate a comprehensive assessment by qualified forensic psychiatrists, incorporating clinical presentation, psychopathological features, social context, and psychosocial factors—particularly in complex cases. The quantitative data from ADL and FSIQ should be regarded as one evidentiary component within this broader and more nuanced evaluation framework.

This study also invites reflection on the ethical dilemmas inherent in SSDC assessments. The concept of “incapacity” itself is not merely a clinical finding but a legal construct with profound implications for personal liberty and legal accountability. The process of obtaining meaningful consent in a forensic context is challenging, as evaluees may not be fully autonomous or may be influenced by the coercive nature of the legal setting. Our reliance on clinical thresholds to define “absent SSDC” must be cautiously interpreted within this framework, ensuring it does not lead to the stigmatization or perpetual disempowerment of individuals with mental disabilities. The assessment should be viewed as a snapshot of their capacity within a specific context, not an absolute judgment on their personhood.

Finally, from the perspective of the overall judicial practice process, the combined application of ADL and FSIQ has established a continuous assessment system from case investigation to forensic identification. This system not only ensures the scientificity and consistency of assessment results, but also minimizes subjective biases in the assessment process through standardized operating procedures, ultimately providing solid technical support for the fair handling of sexual assault cases. In the future, with the further standardization and localization of assessment tools, this joint assessment model is expected to play its unique value in a wider range of judicial practices. However, our model identifies FSIQ and ADL as significant predictors at a group level, their application must be contextualized within a comprehensive clinical and forensic evaluation. This is particularly critical for individuals with schizophrenia, where SSDC cannot be inferred from diagnosis alone but requires detailed analysis of the clinical condition at the time of the offense—including positive/negative symptoms, clinical compensation, relationship to the offender, and capacity to exercise informed will. It is important to note that a key limitation of our retrospective design is the lack of granular data on specific psychotic symptomatology and psychosocial context. Therefore, our quantitative findings should be interpreted as screening indicators that signal the need for deeper phenomenological assessment, rather than as definitive determinants. Future prospective studies incorporating structured clinical interviews are needed to address this gap.

### 4.5 Limitations

This study has several limitations, primarily stemming from its retrospective design based on archived forensic files. This approach prevented us from controlling all variables of interest or standardizing assessment conditions across the five participating institutions, despite all following national guidelines. The cross-sectional nature of the data also means we cannot infer causality or observe the potential fluctuation of SSDC over time, which may be influenced by treatment, rehabilitation, or changes in social context. To address these limitations, we strongly recommend future studies to employ prospective, longitudinal designs. Tracking a cohort of individuals with mental disorders over time would be invaluable for understanding the dynamic evolution of SSDC, identifying critical periods of vulnerability or improvement, and establishing how changes in clinical factors (e.g., FSIQ, ADL) and social factors correlate with changes in capacity. And The sample was predominantly collected from forensic psychiatric institutions in Beijing, Guangyuan (Sichuan), and Suzhou (Anhui), resulting in geographical imbalance that may affect the generalizability of findings - future multicenter collaborations should expand sample size and geographical coverage, particularly including ethnic minority regions and culturally diverse areas to enhance representativeness. Regarding assessment tools, cultural influences were insufficiently considered; subsequent studies should develop culturally adaptive instruments by establishing culture-sensitive frameworks, adjusting evaluation standards for different cultural contexts, and incorporating localized cultural factors into assessment systems, while conducting cross-cultural validation to ensure tool applicability across diverse backgrounds.

Beyond the clinical and psychometric factors examined in this study, it is imperative to consider the broader socio-structural context that shapes the vulnerability of women with mental disorders. Structural violence, manifested through systemic inequalities in access to education, healthcare, and legal protection, can profoundly impair an individual’s resources and capacity for self-defense. Furthermore, the pervasive stigma associated with both mental illness and sexual victimization often silences victims and discourages them from seeking help, thereby diminishing their de facto SSDC. Gender inequality, a cross-cultural issue, also plays a critical role, as it can normalize sexual coercion and undermine a woman’s perceived right to bodily autonomy. Our findings on the predictive power of ADL, which encompasses social functioning and community integration, may indirectly reflect the outcome of these structural factors. Future research should actively integrate quantitative measures of social support, stigma exposure, and gender norms to build a more ecologically valid and comprehensive model of SSDC assessment.

For forensic applications, the current combined assessment protocol inadequately addresses the complexity of real-world evaluations - future work should integrate multidimensional indicators (e.g., micro-expression analysis, verbal demeanor assessment), develop intelligent evaluation systems combining structured scales with unstructured behavioral observations, and establish judicial protection mechanisms linking assessment results to specific legal interventions. Finally, this study focused primarily on adults, lacking specialized research on adolescents and other special populations - subsequent investigations should conduct age-specific studies considering developmental factors and establish tailored assessment systems for different age groups and developmental stages. Furthermore, despite using standardized tools, the SSDC assessment remains a clinical judgment that may be subject to certain biases. Expert subjectivity, though mitigated by training and guidelines, cannot be entirely eliminated. Moreover, while we included participants with various mental disorders, we did not have a granular measure to control for the acute severity of symptoms at the time of the offense, which could be a confounding variable. Future studies would benefit from incorporating structured clinical interviews and symptom severity scales to better control for this confounding effect and further objectify the assessment process.

## Data Availability

Contact the author to obtain their permission. Requests to access the datasets should be directed to 1963347097@qq.com.
